# Human Injury Criteria for Underwater Blasts

**DOI:** 10.1371/journal.pone.0143485

**Published:** 2015-11-25

**Authors:** Rachel M. Lance, Bruce Capehart, Omar Kadro, Cameron R. Bass

**Affiliations:** 1 Code E15 Underwater Systems Development and Acquisition, Naval Surface Warfare Center Panama City Division, Panama City, Florida, United States of America; 2 Pratt School of Engineering, Duke University, Durham, North Carolina, United States of America; 3 Veterans Administration Medical Center, Durham, North Carolina, United States of America; 4 Department of Psychiatry and Behavioral Sciences, Duke University Medical Center, Durham, North Carolina, United States of America; 5 William Beaumont Hospital, Royal Oak, Michigan, United States of America; University of California San Diego, UNITED STATES

## Abstract

Underwater blasts propagate further and injure more readily than equivalent air blasts. Development of effective personal protection and countermeasures, however, requires knowledge of the currently unknown human tolerance to underwater blast. Current guidelines for prevention of underwater blast injury are not based on any organized injury risk assessment, human data or experimental data. The goal of this study was to derive injury risk assessments for underwater blast using well-characterized human underwater blast exposures in the open literature. The human injury dataset was compiled using 34 case reports on underwater blast exposure to 475 personnel, dating as early as 1916. Using severity ratings, computational reconstructions of the blasts, and survival information from a final set of 262 human exposures, injury risk models were developed for both injury severity and risk of fatality as functions of blast impulse and blast peak overpressure. Based on these human data, we found that the 50% risk of fatality from underwater blast occurred at 302±16 kPa-ms impulse. Conservatively, there is a 20% risk of pulmonary injury at a kilometer from a 20 kg charge. From a clinical point of view, this new injury risk model emphasizes the large distances possible for potential pulmonary and gut injuries in water compared with air. This risk value is the first impulse-based fatality risk calculated from human data. The large-scale inconsistency between the blast exposures in the case reports and the guidelines available in the literature prior to this study further underscored the need for this new guideline derived from the unique dataset of actual injuries in this study.

## Introduction

The lethal effects at a distance of underwater blasts have been known at least since the 1820s following the clearance and salvage of the Royal George in Portsmouth harbor in 1829 [1, 2]. Underwater blast injuries to humans were first described as the result of accidental depth charge detonations in 1916 during WWI [3]. Since then, the military and scientific communities have repeatedly called for a realistic injury guideline for underwater blast exposures; however, even as recently as 2001 that need had yet to be fulfilled [4]. There is extensive literature on pulmonary injury and fatality risk assessments for air blast, some of the work driven by potential nuclear weapons exposure [5] and short and long peak overpressure duration military exposure (e.g. [6–8]). More recently, neurotrauma injury and fatality assessments have been derived for blasts in air [9, 10]. Much of this work was based on scaling animal risk assessments to human exposure conditions. Owing to differences in coupling between air/torso and water/torso, it is unclear how these air blast studies may apply to underwater blast injury risk.

Though underwater blasts propagate further and injure more readily than air blasts [11], the current U.S. Navy underwater risk guidelines are not based on actual blast exposure data. Instead, they are based entirely on the untested assumption published in 1943 that since the surface of the water “shreds” (creates a plume) at approximately 3440 kPa (500 psi), the same pressure guideline must apply for tearing the inside of the human lungs [12]. This speculation has propagated through Navy literature since its original publication in 1943, and has never been updated through experimentation, theoretical calculations or any other means [13]. In fact, very few underwater blast injury guidelines have been based on data of any kind, and those guidelines that were based on data are still remarkably inconsistent with each other [14]. This inconsistency is likely caused by the non-ideal experimental setups and lack of appropriate inter-species scaling found in the majority of these experiments [15–19]. Despite the lack of accurate guidelines, military missions frequently expose personnel to underwater blast with an unquantified risk of injury or death. The purpose of this study was to use actual exposure data to create a meaningful underwater blast injury guideline.

## Background

While air blast injuries typically can occur via one of four general mechanisms [20], the increased density, sound speed, and viscosity of water relative to air mean that underwater blast injuries occur almost exclusively as the direct result of overpressure or primary blast. The increased viscosity virtually eliminates the potential for injury from fragments (referred to as secondary injuries) at moderate distances from the charge. Similar to air blast, the gas-containing organs are by far the most affected in an underwater blast exposure. Occasional lesions of the liver occur [21, 22], but the majority of injuries occur in the lungs and intestinal tracts through spalling of epithelium and microvasculature into air spaces [23–26]. The high prevalence and severity of intestinal damage is unique to underwater blast injuries. In addition, most available cases occur near the surface of the water. The importance of proximity to the surface on the resulting injury risk will be discussed in more detail in below.

**Fig 1:**
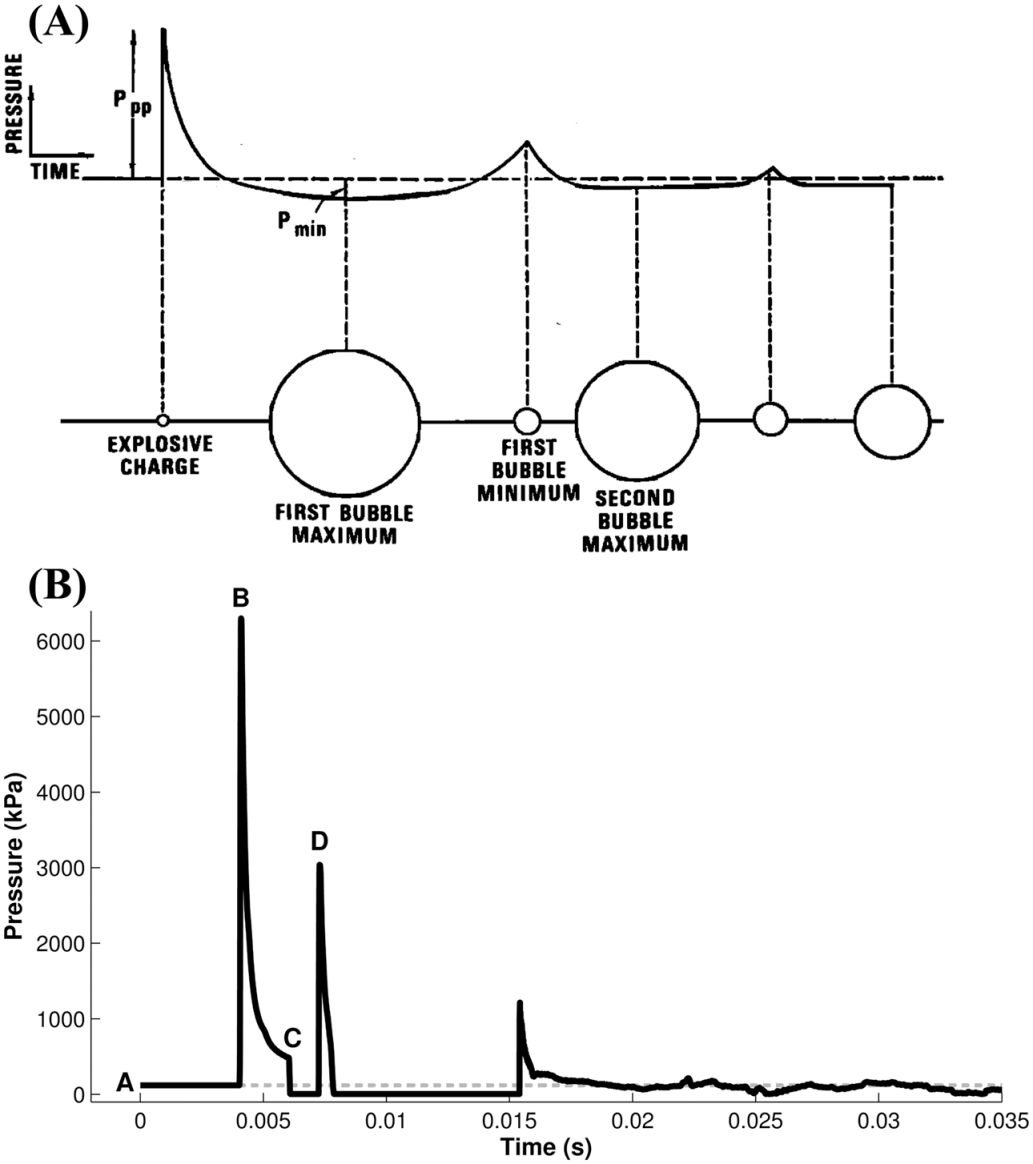
Example pressure-time curves for underwater blasts. (A) Idealized curve. Adapted with permission from Gaspin JB, Goertner JA, Blatstein IM (1979) The determination of acoustic source levels for shallow underwater explosions. The J. Acoust. Soc. Am. 66:1453–1462. [92] Copyright 1979, Acoustical Society of America. (B) Example curve for an underwater blast with surface and bottom reflections. A: ambient pressure. B: peak positive overpressure. C: onset of pressure reduction from surface rarefaction wave. D: secondary peak from bottom reflection.

Unlike for air blast, even ideal underwater blasts may not have a waveform that can be described using a Friedlander-like equation. Underwater blast waveforms are affected by numerous parameters including charge depth, bottom depth, gage depth, bottom reflectivity, and gas bubble fluctuations following detonation. Their effects on the shape of the blast waveform have been investigated exhaustively since the early 1940s and some are still the subject of active research [27–32]. While several literature resources provide simple equations to describe the peak pressure and initial decay of the shock wave [29, 33, 34], these equations all assume an exponential decay to the waveform. This assumption is only valid for a single time constant of decay, and does not incorporate the subsequent waveform or the actions of the gas bubble. Similarly, scaling laws are available for ideal blasts underwater [35]. However, blasts can cause serious injuries and fatalities even in extremely low pressure ranges [36]; the limited data that are available to validate these laws in these pressure ranges suggest that the laws overestimate the impulse of exposure [37]. Though the full details of underwater blast physics are beyond the scope of this publication, two main points are important to this study: 1) no research group has ever identified a Friedlander-like equation that accurately describes a generalized underwater blast waveform and 2) the surface of the water reflects a tension, or rarefaction, wave back down into the body of water that decreases the pressure of the primary waveform wherever the two intersect. This rarefaction wave can result in a dramatic decrease both in peak pressure and in overall impulse for measurement points near the surface of the water. These decreases play an important role because the majority of human exposures to underwater blast have occurred at or near the surface of the water. Neither the exponential decay models nor the scaling laws discussed can account for the negative pressure reflection off the surface. Like air blasts, positive-pressure waves in underwater blasts are reflected off of surfaces with higher densities such as structures or the ocean bottom, but this effect is uncommon in human exposures unless the exposure occurs in shallow water or an enclosed space. Because of these complexities, descriptive parameters like peak pressure and impulse are often difficult to predict without advanced computational modeling. Fig 1 shows idealized examples of underwater blast waveforms with identification of their various components.

The overall intensity of a blast exposure, which is responsible for injury severity, depends on more than one physical parameter such as peak pressure and pressure duration that contribute to the observed pressure or impulse time history. For ideal air blasts, the Friedlander waveform allows an indirect but comprehensive description of blast intensity through only two parameters, often peak pressure and positive phase duration. Many currently-available air blast injury criteria use these two parameters to describe exposure and therefore injury risk (cf. 5, 6). These same criteria cannot be used for the complex waveforms from underwater blasts because the entire shape of the curve is much more variable and a positive phase duration value is often difficult to determine. An actual impulse value is therefore a more comprehensive description of the overall blast waveform. The difficulty in precisely calculating or predicting impulse has led most researchers to prescribe a guideline based on range or peak pressure, even though these factors have long been thought to be insufficient [14, 18, 28, 38–40].

## Methods

### Injury ratings

In contrast with air blast, there is a very limited amount of well-characterized experimental animal or human exposure data for underwater blast [17, 18, 22, 41, 42]. However, during WWII at least as many military casualties were incurred from underwater blast as from air blast [43]. Hundreds of case reports of underwater blast exposure were published by military physicians, and many of these case reports contain extensive information about the scenario creating the blast exposure [3, 15, 24, 39, 44–61]. Much of this literature is based on experiences in WWII when sailors in the water were exposed to blasts from depth charges, either from enemy vessels or from primed charges that detonated as their own ships sank. In addition, a handful of more recent human experiments and isolated blast incidents have been published with details of the exposures [17, 21, 26, 40, 62–65]. The resulting database included 475 human exposures at various ranges from the blasts, making it the largest underwater blast injury database compiled to date.

The injuries described by the physicians ranged from mild abdominal discomfort and coughing to near-instantaneous fatalities. Many case studies contain comprehensive anatomical information, but frequently a detailed description of symptoms was the only medical information provided. Since modern injury rating systems do not rely upon symptoms alone, rating systems for both pulmonary and abdominal injuries were developed that included seven ordinal levels of severity. These numerical severity ratings were based on the reported symptoms, surgical findings, and autopsy reports. Severity estimates were derived from a collaboration of physicians and biomedical engineers including a colon and rectal surgeon with decades of experience treating intestinal abnormalities. For comparison, the injury ratings were associated with the Abbreviated Injury Scale (AIS) trauma injury scales for both pulmonary injuries [66] and abdominal injuries [67]. The ratings were designed to minimize the effect of differences in medical care across the cases, relying upon initial presentation of the patient rather than long-term prognosis. The rating scales are shown in Tables 1 and 2.

**Table 1:** Injury rating scale for abdominal injuries from underwater blast exposures.

ABDOMINAL INJURY SCALE			
Rating	Severity	Symptomatic Scale	AIS Scales for Small Bowel/Colon/Rectum [67]
0	None		
1	Minor	X-ray evidence; OR mild hemorrhaging; OR discomfort/pain; OR localized rigidity; NO general rigidity	Contusion or hematoma without devascularization; OR partial-thickness laceration without perforation
2	Moderate	Moderate hemorrhaging; OR discomfort/pain/general rigidity indicative of perforations deemed not to require surgery based on standards of treatment at time of injury^+^	Laceration <50% of circumference
3	Serious	Severe hemorrhaging; OR perforations severe enough to warrant surgery based on standards of treatment at time of injury^+^	Laceration ≥50% of circumference without transection
4	Severe	Multiple or unusually large perforations, possibly severe enough to cause death in 1943 but likely treatable by modern medical practices	Transection of small bowel/colon; Full-thickness laceration of rectum with extension into peritoneum
5	Critical	Untreatable in 1940s, would still likely be untreatable now; primarily palliative measures	Transection with segmental tissue loss in the small bowel/colon; OR devascularized segment in the small bowel/colon/rectum
6	Maximum	Fatality within 30 minutes of exposure	Maximal (currently untreatable)

The AIS Scale is provided only as a reference to compare severity of rankings, and is not intended to suggest common methods of treatment between the two independent scales.

^+^Modern medical standards mandate surgery for any size intestinal perforation. In contrast, surgery in the 1940s carried a large risk of infection, and antibiotics were far less available/effective. The decision to operate was used to assess, retrospectively, the severity of the patient’s symptoms upon presentation, not to denote an acceptable modern medical standard of care.

**Table 2:** Injury rating scale for pulmonary injuries from underwater blast exposures.

PULMONARY INJURY SCALE			
Rating	Severity	Symptomatic Scale	AIS Pulmonary Scale [66]
0	None		
1	Minor	Some x-ray evidence but asymptomatic	Contusion (unilateral <1 lobe)
2	Moderate	Coughing; OR shallow breathing	Contusion (unilateral whole lob); OR laceration (simple pneumothorax)
3	Serious	Mild hemoptysis; OR difficulty breathing	Contusion (unilateral >1 lobe); OR laceration (persistent >72hrs, airleak from distal airway); OR hematoma (nonexpanding intraparenchymal)
4	Severe	Severe symptoms, treatable by modern medical practice, possible recovery or fatality	Laceration (major airway leak); OR hematoma (expanding hematoma); OR vascular (primary branch intrapulmonary vessel disruption)
5	Critical	Severe cyanosis; OR severe hemoptysis; likely untreatable by modern medical practice; typically fatal	Vascular (hilar vessel disruption); OR multilobar lung laceration with tension pneumothorax
6	Maximum	Fatality within 30 minutes of exposure	Maximal (currently untreatable)

The AIS Scale is provided only as a reference to compare severity of the rankings, and is not intended to suggest common methods of treatment between the two independent scales.

After the rating scales were developed, the injuries in the database were independently assigned a numerical severity by three different reviewers: a former-Army physician with prior blast trauma experience (BC), a biomedical engineering professor with extensive experience in blast research (CB), and an engineering PhD student blast researcher (RL). Each reviewer blindly and independently rated each injury according to the scales and the Cohen’s kappa coefficient was calculated to determine inter-rater reliability between the physician and the experienced PhD reviewers (0.42 for the abdominal scale, 0.54 for the pulmonary scale). The PhD student’s ratings were used only as a tiebreaker to determine a final injury value for cases with conflicting reviewer values. The final Cohen’s kappa coefficients between the physician reviewer’s values and the final values were 0.65 and 0.70 for abdominal and pulmonary injuries respectively, and the coefficients for the experienced PhD reviewer were 0.71 and 0.77 respectively. These high kappa values indicate that the scale developed is sufficiently detailed to consistently describe the level of injury for the cases in the database.

### Blast assessment

Many of the case reports contained sufficient detail to completely reconstruct the exposure scenarios, including charge type and estimated distance from the explosion center. Some of the publications issued during WWII did not contain detailed information on charge types because the information was still considered sensitive, but the charge types could be determined retroactively based on incident dates, vessels involved, locations, and personnel nationalities. Most of the exposures were depth charges, which had either a preset detonation depth or a very limited number of user-selectable detonation depths. Based on the depth charge model and the type of warfare being conducted when the vessel was sunk, the detonation depth could usually be determined fairly conclusively. If a case did not contain sufficient information to determine all of the scenario parameters, that case was eliminated from the final dataset.

Though there are no simple theoretical models for underwater explosions, they can be accurately modeled using finite-element and finite-volume methods. These computational programs can account for the many factors that complicate underwater blasts [68, 69]. One of the most prominent pieces of modeling software is the US Navy’s DYSMAS (Dynamic System Mechanics Advanced Simulation) hydrocode, which uses the Gemini Eulerian solver to model the pressures resulting from underwater blasts. This software has been extensively validated and shown to accurately mathematically reproduce the effects of underwater blasts, even in complex environments [70–75]. DYSMAS provided general pressure time histories based on ranges and explosives from the case literature to correlate with the observed injuries from underwater blast. Using this software, the exposures could be accurately modeled to include all known confounding variables such as bottom depth, proximity to the surface, and varying degrees of bottom reflectivity.

Once the exposures were reconstructed, the US Navy’s Gemini Eulerian solver was used to compute the peak pressures and impulses at the reported location of each blast injury victim. For blast victims immersed from the neck down at the surface of the water, the lungs were approximated as 10 cm beneath the surface and the lower abdomen was approximated as 30 cm beneath the surface. Though these differences are generally a much shorter length scale than the horizontal separations from the charge, the resulting pressure time histories were sensitive to the distance of each organ system under the water. For example, this 20-cm distance typically yielded peak pressure and impulse values that differed by a factor of 2–3 owing to the rarefaction wave reflecting off the surface of the water. The abdominal exposures were therefore significantly different from the pulmonary exposures for the same person (p<0.001 for both peak pressure and impulse values). Orientation in the water was reported in some case studies, but was generally not reported with enough frequency or descriptive detail to use it to determine vertical position on a finer scale.

The sensitivity of the results to variations in the reported surface distances was evaluated by varying a subset of sample cases by ±20% and evaluating the magnitude of the change in the exposure levels seen by the blast victims. Before the age of radar ranging, accuracy of distance estimation by eye for trained observers measured as approximately 5–10% at ranges similar to those used in this study [76]. More recent reports include range estimation errors of approximately 10% for untrained ground soldiers [77] and less than 20% estimation error in assessing distance to boats [78]. More recent studies with military personnel include Wright (1995) (9–13% estimation error) [79], Lampton et al. (1995) (<5% error at 10 m range) [80], and Sun (2004) (<10% error for several tasks) [81].

These conservative distance error estimates were incorporated into a sensitivity analysis on the distances from the charges and resulting impulse values. Based on a potential 20% distance variation, a set of 100 risk analyses was performed for each risk assessment developed in this study with each impulse value perturbed by a Gaussian random number with zero mean and a standard deviation representing the calculated impulse at ±20% distance variation. Because the variation of impulse is not symmetric with distance, closer distances have larger impulse increases than proportionally larger distances have impulse decreases.

### Protection

Throughout the historical literature, authors repeatedly state their beliefs that life preservers should help mitigate the injurious effects of underwater blast in the lungs [12, 82]. These beliefs were stated without any evidence and were contended even as early as their initial publication in 1943 [57]. Recent investigation shows that modern personal protection in air blast offers substantial protection to the lungs [83], potentially increasing the relative occurrence of abdominal injuries [84]; however, the protection studied covers more of the chest and is much closer-fitting than the “Mae West” or belt-style life preservers of the time period.

While some testing has been performed to determine the protective effect of life preservers, the tests have either been inconclusive or determined that the area of protection needs to cover the entire torso to be effective [85, 86]. In the absence of experimental data, statistical analysis of the exposure dataset was performed to determine if wearing a life preserver provided a protective effect. Since the “life preserver” dataset was relatively small, the protective effect of life preservers was investigated by comparing relative injury levels between abdomen and pulmonary systems of protected and unprotected wearers. If life preservers provided protection to the lungs, then for a comparable level of pulmonary injury the abdominal injuries for personnel wearing life preservers should have been worse than for personnel who were not wearing life preservers.

Abdominal injury severity was plotted as a function of pulmonary injury severity for the 187 injury data points reporting life preserver use. Cases with no reported injuries were eliminated from this phase of the analysis. The personnel not wearing life preservers and the personnel wearing life preservers were treated as two completely separate groups, and a linear model was fit to each set of data. The slopes of these lines were compared, and statistical significance was evaluated to determine the effect of life preservers. An ANCOVA analysis was also performed on the data, as well as Mann-Whitney U tests at each level of lung injury severity. No significant effect could be detected by any of the tests, so life preserver use was eliminated as a statistical variable. The results of these analyses are presented below.

### Survival analysis

The injury levels for each organ system were separated into one of three groups: non-injury, injury, and fatality. Injury severity levels 0 and 1 were grouped as “non-injury” because level 1 injuries are, by definition, asymptomatic. Injury levels 2–4 were grouped as “injury” since the medical treatments for these levels would be similar and the patients would have a good chance of survival. Injury levels 5 and 6 were grouped as fatal because, even by modern standards, these patients would likely result in fatalities. While several level 4 cases resulted in fatalities, they were grouped as injuries because many of these cases died from infection and would likely be treatable with modern antibiotics, imaging, and surgical techniques.

Parametric survival analyses were performed for each organ system using Minitab (Version 17, Copyright ©2014, State College, PA, USA) resulting in four total impulse-based risk functions for blast exposure. The risk functions were in the form of a Weibull distribution, shown as Eq 1.

$$f(I)=\frac{\beta }{\eta }{\left(\frac{I}{\eta }\right)}^{\beta -1}e^{{-\left(I/\eta \right)}^{\beta }}$$ (1)

When calculating the injury risk functions, severity levels 0–1 were considered as right-censored uninjured and levels 2–6 were considered interval-censored injuries, with possible injurious values between 0 and the calculated blast impulse. The same procedure was followed to determine the fatal risk functions, with injuries ≤4 considered nonfatal and injuries level 5–6 considered fatal. Cases that gave a range of possible distances were considered right-censored from the minimum possible exposure if an injury or fatality did not occur and interval-censored between 0 and the maximum possible exposure if an injury or fatality did occur.

The probability of injury or fatality can be calculated using the cumulative distribution function for the Weibull distribution. This function is shown as Eq 2, where F signifies the risk of injury or fatality.

$$F\left(I\right)=1-e^{{-\left(I/\eta \right)}^{\beta }}$$ (2)

### Range predictions

To provide safe distance estimates for underwater blast, the 20% and 50% risk values from the pulmonary and abdominal injury and fatality curves were translated into a function of range (R) vs. charge weight (W) using the experimentally-validated scaling law for impulse (I) shown in Eq 3. The impulse values for injury were lower for either the abdominal or pulmonary risk functions, depending on the percent risk, but the impulse values for fatality were always lower for the pulmonary risk functions. Overall injury or fatality risk by range is calculated by the organ system that gives the highest risk at the lowest impulse values.

$$I=kW^{1/3}{\left(\frac{W^{1/3}}{R}\right)}^{\alpha }$$ (3)

Values for k and α corresponding to TNT were used in this analysis (k = 6,698; α = 0.94)[35]. While TNT itself is rarely used for modern military purposes, it remains the standard for comparison of charge strengths. Owing to the rarefaction wave reflected off the surface, Eq 3 describes only fully immersed cases that are deep enough to avoid the protective effect of the surface. Swimmers on the surface would be safe at greater distances than submerged swimmers because of the reduction in pressure from the reflected tension.

## Results

### Sensitivity Analysis

The means for all of the ensemble perturbed risk calculations are within the 95% confidence interval for the calculated risk functions for lung and abdominal injury and fatality at the 50% risk levels. The mean 100 random ensemble value for impulse at 50% risk for lung injury was 269±11 kPa-ms, for lung fatality was 422±138 kPa-ms, for abdominal injury was 221±20 kPa, and for abdominal fatality was 839±77 kPa. It was therefore concluded that the estimated distances served as valid approximations for calculation of survival curves.

### Protection

**Fig 2:**
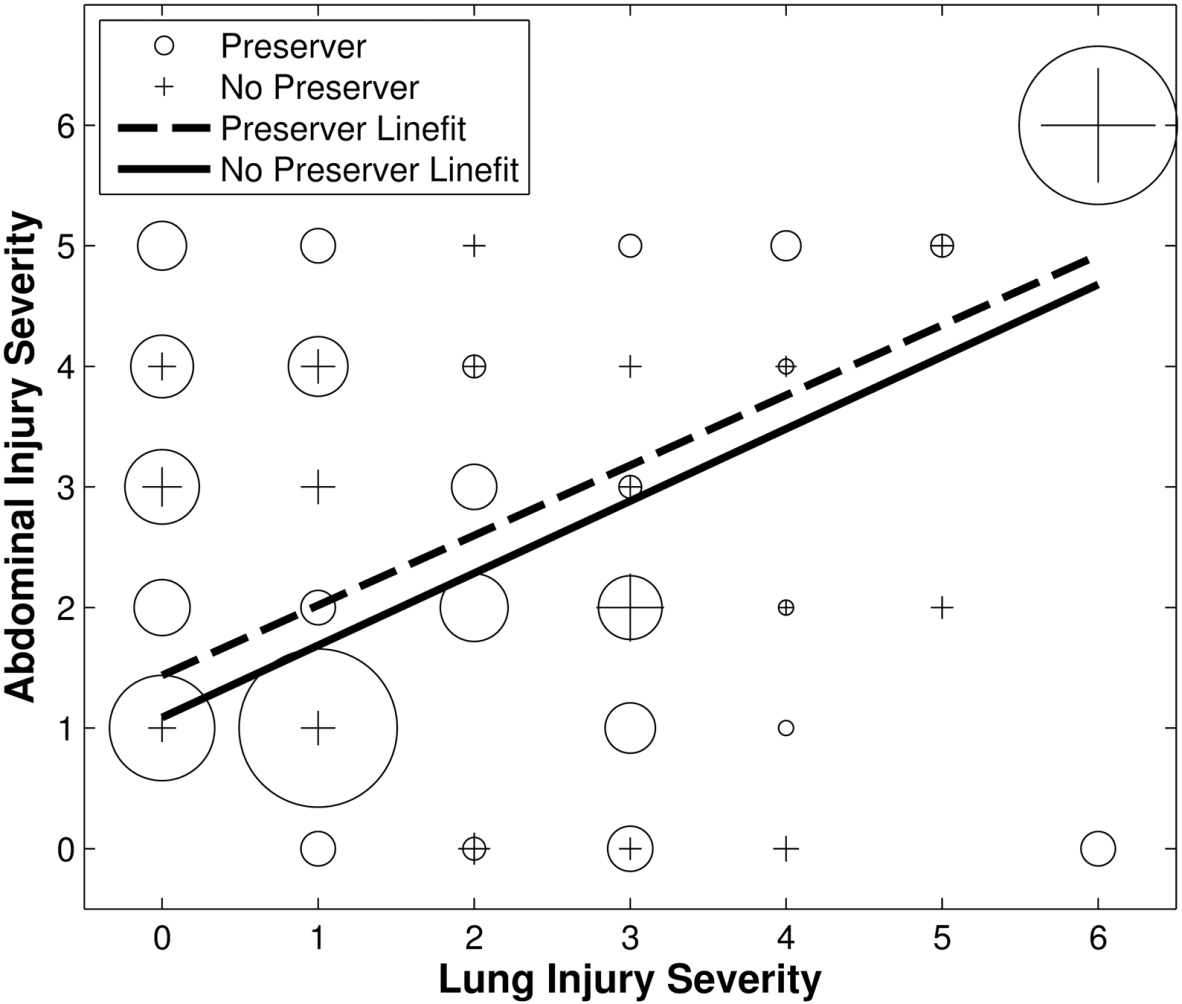
Abdominal injury severity as a function of pulmonary injury severity. For two groups wearing and not wearing life preservers at the time of blast exposure. Marker size is proportional to the number of data points.

The injury data and regression fit lines for the life preserver analysis are shown in Fig 2. Both regression lines are acceptable fits to the data (slope = 0.58, intercept = 1.44, R^2^ = 0.63 with life preserver; slope = 0.60, intercept = 1.09, R^2^ = 0.55 without life preserver). The sizes of the markers in Fig 2 are proportional to the number of data points at those locations.

While the R^2^ values are moderate, the p-values (<0.0001) for the slopes of both lines confirm that there is a statistically significant increase in abdominal injury severity with increasing pulmonary injury severity. This result is not surprising, since more severe injuries to both systems would logically result from an overall higher blast exposure. However, there is no significant difference between the slopes of the lines (p>0.46). Similarly, ANCOVA analysis confirmed the dependence of abdominal injury on lung injury but showed no relationship with life preserver use (p_lung injury_<0.0001; p_jacket use_>0.31; p_interaction_>0.90). In addition, Mann-Whitney U tests were performed on the distribution of abdominal injuries within each ranking category of lung injury. None of the seven separate Mann-Whitney U tests showed a statistically significant difference between the two groups. Life preserver use was therefore eliminated as a variable in injury risk.

### Survival analysis

**Fig 3:**
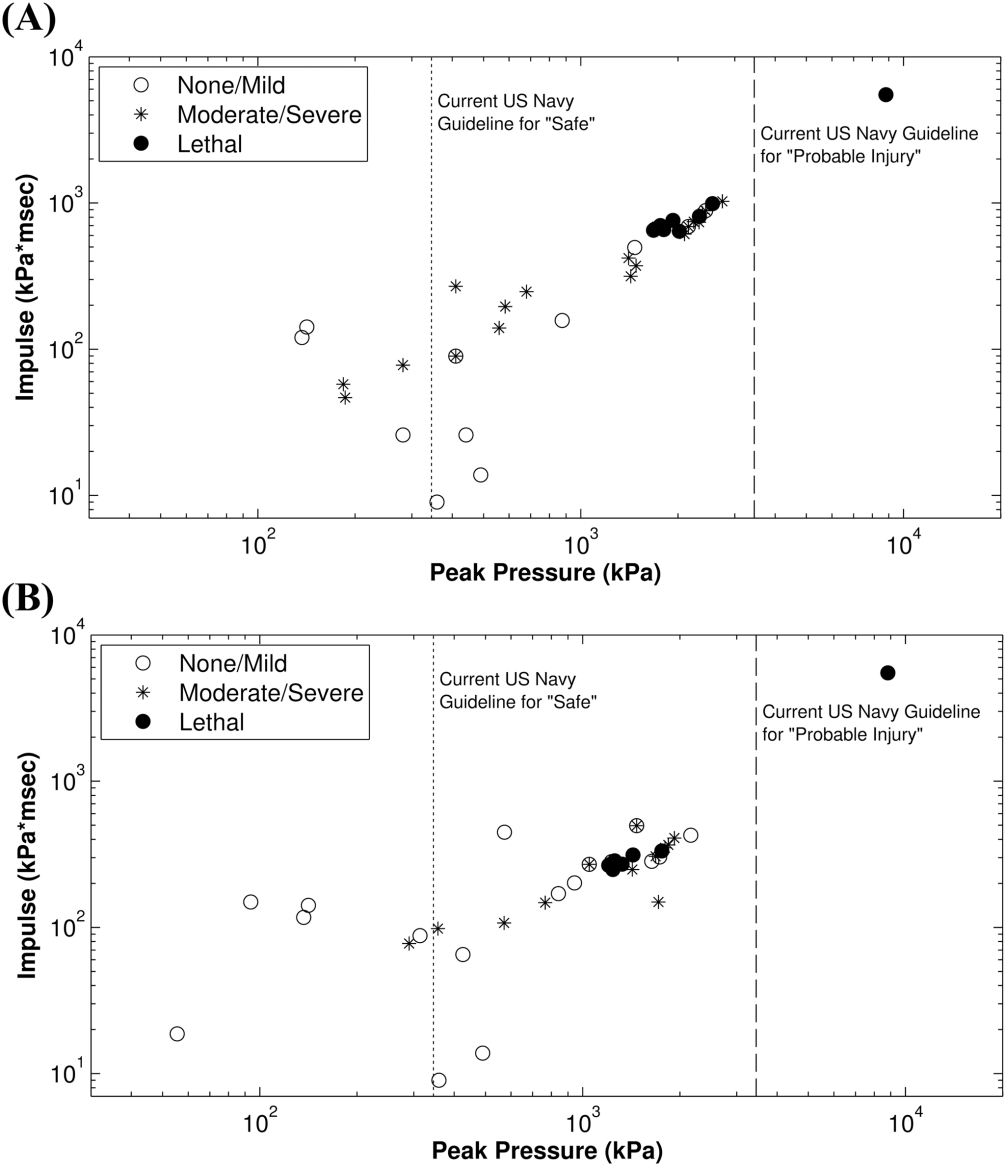
Abdominal and pulmonary injuries. (A) Abdominal injuries. (B) Pulmonary injuries. Injuries plotted against peak pressure and impulse of exposure. Dotted and dashed lines represent current US Navy guidelines for “safe exposure” (50 psi, or 345 kPa) and “probable injury” threshold (500 psi, or 3447 kPa).

Fig 3a and 3b show the injury data for both organ systems plotted against peak pressure and impulse of exposure. For simplicity, cases with a range of possible distances are shown plotted at the exposure values corresponding to the mean distance. These results are compared with the current US Navy guidelines for safe exposure levels and probable injury threshold.

**Fig 4:**
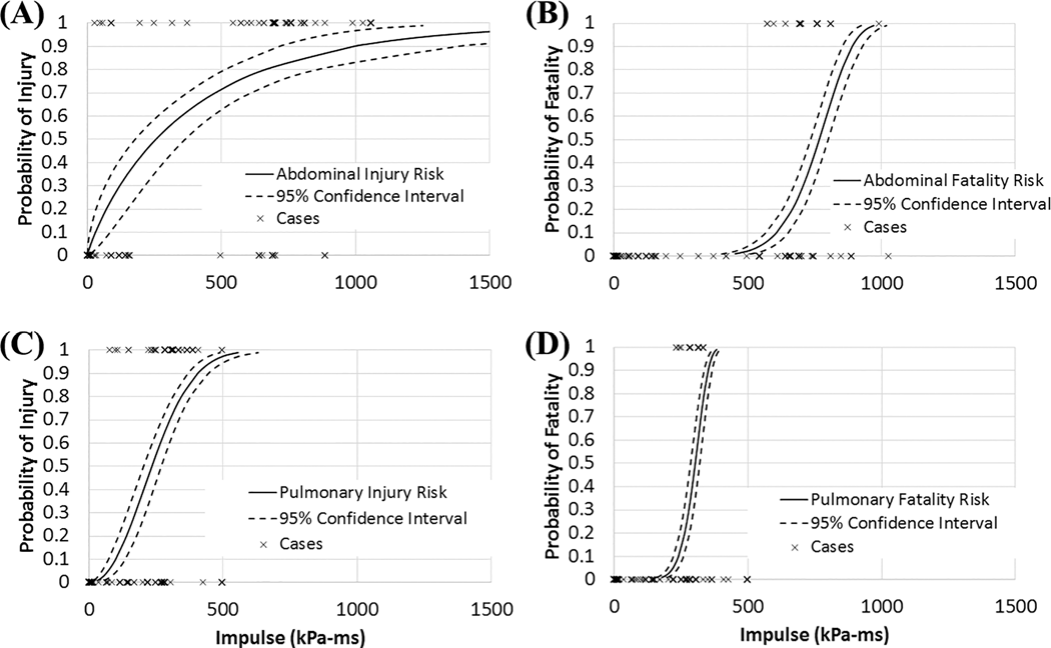
Calculated injury and fatality risk functions. (A) Abdominal injury risk function (Anderson-Darling test statistic = 1.58) (B) Abdominal fatality risk function (A-D = 11.96) (C) Pulmonary injury risk function (A-D = 5.60) (D) Pulmonary fatality risk function (A-D = 22.90)

Fig 4 shows the pulmonary and abdominal injury and fatality risk functions as computed by Minitab. The coefficients of the equations are shown in tabular form in Table 3, and the equation to calculate risk is shown as Eq 2. Table 4 shows calculated impulse values for 10% and 50% risks.

**Table 3:** Risk function constants.

	Injury Risk Function	Fatality Risk Function
**Abdominal**	β = 0.885; η = 386.6	β = 8.048; η = 802.5
**Pulmonary**	β = 2.197; η = 277.5	β = 8.068; η = 316.5

**Table 4:** 50% risk values.

	Injury Risk (kPa*ms)		Fatality Risk (kPa*ms)	
	10%	50%	10%	50%
**Abdominal**	30 +56/-19	256 +110/-77	607 ± 44	767 ± 33
**Pulmonary**	100 ± 36	235 ± 35	239 ± 20	302 ± 17

### Range predictions

**Fig 5:**
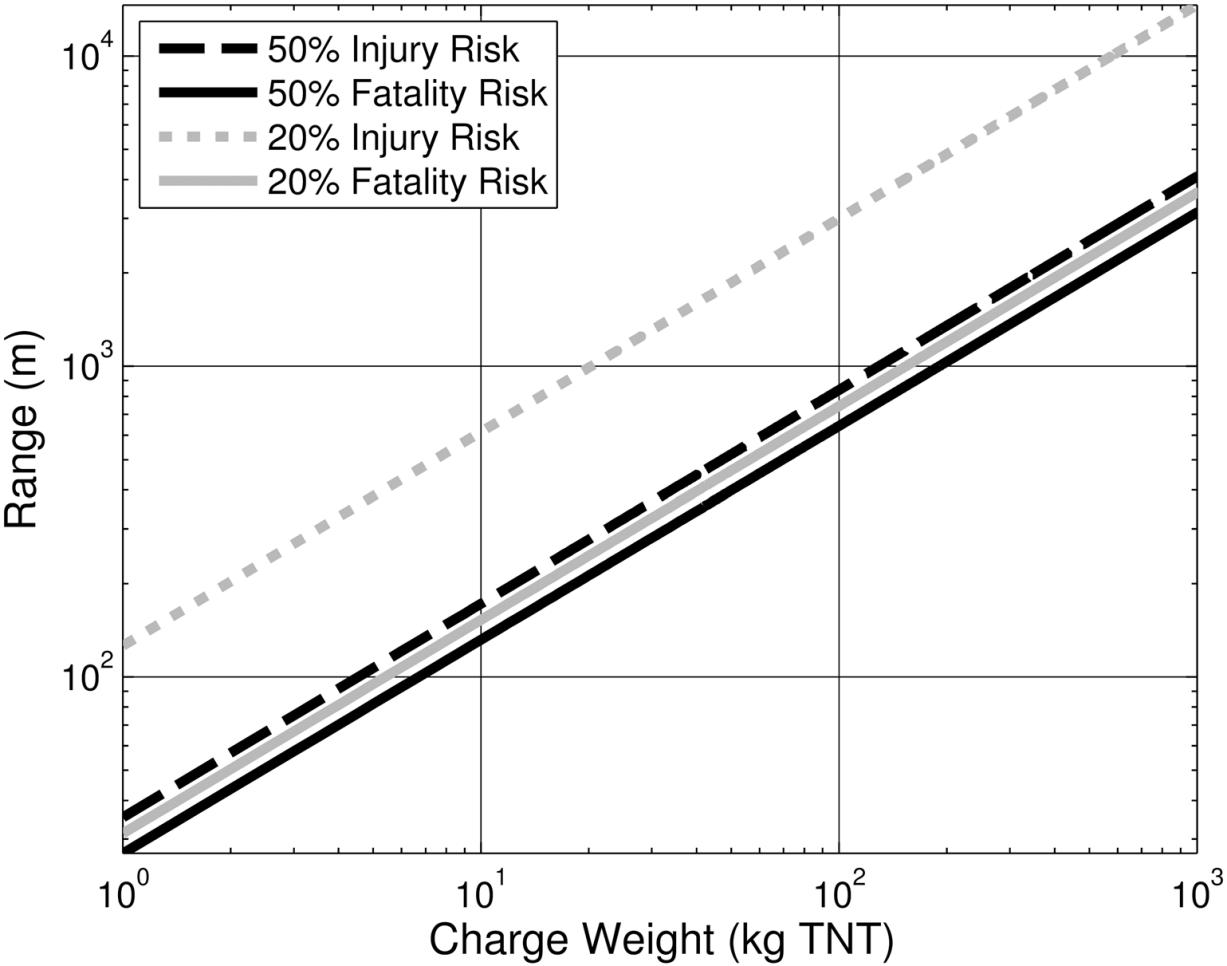
Predicted range for immersion injuries as a function of charge weight. 20% and 50% injury and fatality risk curves.

The calculated range guidelines for immersion for injuries and fatalities are shown in Fig 5. Ranges were calculated via Eq 1, rearranged to solve for R. The corresponding constants remain unchanged (k = 6,698; α = 0.94)[35]. The two scaling constants for TNT, k and α, were converted to Metric values from those found in Ref [35]. These curves predict ranges based on impulse for ideal explosives that have been converted to a TNT standard. For a non-ideal explosive with a higher relative impulse value (e.g., aluminized charges), these range predictions will likely underestimate safe range. However, these scaling laws may overestimate required range for shock waves with less than roughly 133 kPa peak pressure (74). In addition, these scaling laws apply only to ideal, fully-immersed cases without significant bottom reflection. Calculation of ranges in non-ideal conditions requires the use of more complex modeling techniques.

## Discussion

The current US Navy 500 psi guideline for ‘probable injury’ (dashed lines, Fig 3a and 3b) is qualitatively different than the results of the injury assessments from this study. *The largest grouping of human fatalities from underwater blast exposure has exposure levels that are lower than the US Navy ‘probable injury’ guidelines*. The safety guidelines developed by Richmond et al. [17, 87] are the most meticulously-developed standards to date, but even these were not based on human injury data. Instead, they were developed to assert safe levels only, and were based on extrapolations from animal data that were then given a factor of safety. In addition, the gauges in Richmond’s experiments were located 30 cm below the surface with vertical subjects immersed to the neck, so the gauges did not measure the exposure levels seen by organs closer to the surface of the water. The exposure levels closer to the surface of the water can be significantly different from those as deep as 30 cm (p<0.001 for a depth of 10 cm), as discussed in detail in the section Methods: Blast assessment. Richmond et al. attempted to remedy this experimental flaw by retroactively calculating exposure values at shallower depths; however retroactive calculations in a region sensitive to small changes can never be considered as reliable as experimental measurements.

**Fig 6:**
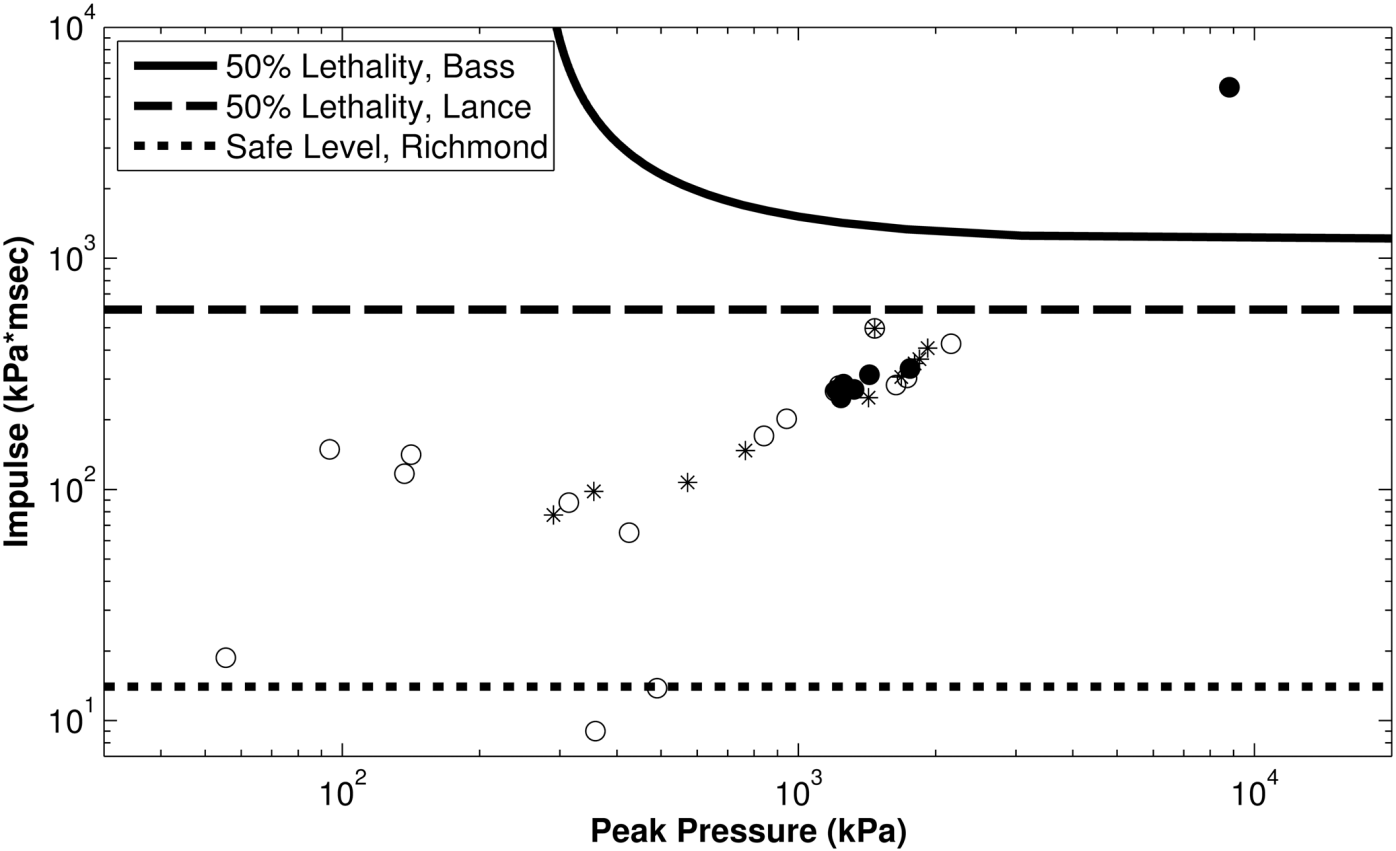
Blast Injury Guidelines. Plotted with the pulmonary data from Fig 2b.

The results of this study are compared directly with air blast fatality risk curves of Bass et al [6] as a function of peak pressure and impulse using a Friedlander approximation (Fig 6). For a blast peak pressure of 1,800 kPa, the corresponding ideal blast in air with a duration of 2 ms would have an impulse of 1,325 kPa*ms and an approximately 50% risk of fatality. At this impulse level, this study predicts an over 99% chance of fatality for pulmonary and abdominal injuries. An example Friedlander blast at the threshold for injury in air (P_max_ = 703 kPa, duration = 2 ms, impulse = 517 kPa*ms) would have an approximately 72% chance of injury in water. This is consistent with the biomechanics of transmission of blast to the chest in air compared with in water. It is expected that water transmission will result in better coupling to the chest because of the relative impedance values of air and water, leading to lower impulse values for injury risk. Comparing Richmond’s experiments with the current study, Richmond predicts a safe level of 14 kPa*ms impulse for swimmers, while this study finds a 1% injury risk level at 34 kPa*ms (95% confidence interval 20–59 kPa*ms) for the chest and 2 kPa*ms (95% confidence interval 0.3–15 kPa*ms) for the abdomen. Since minor chest and abdominal injuries are unlikely to be diagnosed relative to major injuries, the results of this study and Richmond’s guideline are similar in magnitude and are consistent.

Previous investigators have suggested that impulse is a better correlate with human injury than peak pressure, but Fig 3a and 3b show that peak pressure and impulse are very highly correlated with each other for this dataset. Statistical testing also concluded that either could be used to accurately predict injuries for this dataset, in contrast with the assertions of previous investigators. Logically, *both* peak pressure and impulse may be necessary to accurately predict injury risk, similar to the use of both peak pressure and overpressure duration to predict injury risk in air blast. The dataset used in this study, however, almost exclusively contains exposures from ideal high explosives at the surface of an open body of water. Ideal explosives show a close correlation between peak overpressure and impulse values, so while both variables were determined to be predictive of injury risk when used independently, this dataset was not sufficient to separate their relative contributions in a statistically meaningful fashion. In an enclosed space, or with non-ideal explosives, peak pressure and impulse are not always as simply correlated. Experience with damage to underwater structures and pathobiology of air blast at long durations indicate that impulse should be a sensitive predictor of injury severity cf. [88, 89], but it is difficult to demonstrate this without additional data from a wider variety of exposure types.

*From a clinical point of view*, *the results emphasize that blast pulmonary and gut injuries may occur at great distances in water from the source of an explosion*. For instance, using conservative scaling laws for a fully-immersed case, there is a 20% risk of pulmonary/gut injury at 1 km from a 20 kg source (crossref Fig 5). This result suggests that injury may occur in the water at quite long ranges compared with air blast (e.g. [6–8]). This increase in range is relevant in both civilian and military settings. The presence of potential blast pulmonary and gut trauma may be particularly underappreciated in civilian settings (existing civilian reports include [63, 90] and others). It is especially important to appreciate the distance from the source at which such injuries may occur, and that there may be long term sequelae from such events at much larger ranges than implied by air blast experience cf. [91].

## Conclusion

This study provides the first underwater blast injury risk functions based on human data. The substantial difference between current US Navy guidelines and the available human data emphasizes the need for more realistic underwater blast guidelines. The current US Navy guideline for “probable risk of injury” is at a peak pressure value higher than most of the fatalities evaluated in this study. The guidelines adopted from Richmond’s experiments provide an impulse value for safe underwater exposure, but do not provide a conversion from impulse to range. The Richmond guidelines also do not provide information about risk of injury or fatality if personnel are within the recommended range. Using the guidelines published in this study, for the first time military operators can reasonably estimate the risks of injury or death from underwater blast exposure.

Previous clinical literature suggested that the intestinal tract was more vulnerable to injury than the lungs in underwater blast. The majority of this literature consists of medical case reports that are limited to qualitative analyses of the injuries [23, 45, 61, 64], but this assertion also appears in research documents used to determine current US and UK military safety policies [28]. However, this study demonstrated that the abdominal cavity is not more vulnerable; instead, it is exposed to substantially higher levels of blast when the victim is at the surface. Since the majority of historical exposures have occurred at or near the surface, the frequency of severe abdominal injuries has remained subject to this misinterpretation. Our study emphasizes the large distances at which pulmonary and gut injuries may occur. These large distances, while often not appreciated in military and occupational practice, are important for diagnosing potential blast injuries following exposure.

This model has several limitations, primarily based on its use of reconstructions based on historical data. While the DYSMAS hydrocode has been extensively validated, computational reconstruction will always introduce uncertainty compared with real-time measurement of values. In addition, the ranges provided were largely self-reported. Distressed sailors abandoning a sinking ship while swimming rapidly may provide only a gross estimation. At the beginning of the model development, this shortcoming was a concern and was extensively tested. Based upon the sensitivity analysis presented in the Methods and Results sections, it was concluded that reasonable variations in the distance estimates did not lead to any significant alterations in the calculated results. Proximity to the surface could provide an additional complicating factor, especially for the pulmonary risk curves. The lungs were estimated to be 10 cm below the surface of the water; however, the organs span a vertical range broad enough for different exposure values at the proximal and distal boundaries when at this shallow depth of immersion. While 10 cm is a realistic mean value, the high variability near the surface could introduce an additional element of uncertainty into the results and reinforces the need for prospective validation.

This study is limited to ideal pressure profiles in open water. Future work may include long-duration, high-impulse explosive types and closed-environment data if available. This type of data would serve to better separate the influences of impulse and peak pressure for a wider range of applicability.

## Supporting Information

S1 Table Human model dataset. This Microsoft Excel spreadsheet contains all the data used to complete this analysis. Included in this file are the data for the survival analysis and risk curve generation, as well as the data (separated for convenience) for the life jacket and sensitivity analyses.
(XLSX)
